# Clofarabine-based reduced intensity conditioning regimen with peripheral blood stem cell graft and post-transplant cyclophosphamide in adults with myeloid malignancies

**DOI:** 10.18632/oncotarget.26083

**Published:** 2018-09-11

**Authors:** Patrice Chevallier, Pierre Peterlin, Alice Garnier, Amandine Le Bourgeois, Beatrice Mahé, Viviane Dubruille, Nicolas Blin, Cyrille Touzeau, Thomas Gastinne, Anne Lok, Yannick Le Bris, Marie C. Béné, Steven Le Gouill, Philippe Moreau, Thierry Guillaume

**Affiliations:** ^1^ Hematology Department, CHU Hotel-Dieu, Nantes, France; ^2^ Hematology/Biology Laboratory, CHU Hotel-Dieu, Nantes, France

**Keywords:** allogeneic, clofarabine, post-transplant cyclophosphamide, haplo-identical, PBSC

## Abstract

**Background:**

The Baltimore reduced-intensity conditioning (RIC) regimen using high-dose post-transplant cyclophosphamide (PTCY) is considered as a standard of care for haploidentical allogeneic stem cell transplantation (allo-SCT). However, it is associated with relatively low survivals and high incidence of relapse, especially when considering myeloid malignancies.

**Results:**

This retrospective study included 36 adults (males *n* = 18; median age: 60.5 years old; haplodonors *n* = 27; matched donors *n* = 8) with myeloid malignancies transplanted between March 2014 and March 2017 at the University Hospital of Nantes. Very encouraging results were observed with a 18-month overall survival (OS), disease-free survival (DFS) and relapse incidence (RI) of 72% ± 7.5%, 63.8 ± 8%, and 25 ± 6% respectively, and a GVHD relapse-free survival (GRFS) of 52.6 ± 8%. In univariate analysis, there were no differences regarding 18-month survivals between patients allografted: i) for acute myeloid leukemia vs myelodysplastic syndrome (OS 70 ± 11% vs 69.2 ± 13%, *p* = 0.3; DFS 64.7 ± 11% vs 61.5 ± 13%, *p* = 0.65), or ii) with haplo-identical vs other donors (OS: 66.2 ± 9% vs 88.8 ± 10.4%, *p* = 0.16; DFS 59 ± 9.5% vs 77.8%, *p* = 0.6).

**Conclusion:**

The “Clo-Baltimore regimen” is safe and feasible and provides good survivals for patients with myeloid malignancies and haplo-donors.

**Methods:**

Here, we report a variant of the Baltimore regimen, where 1) fludarabine was replaced by clofarabine, 2) bone marrow was replaced by peripheral blood stem cells, and 3) tacrolimus was replaced by cyclosporine, in a “Clo-Baltimore regimen”.

## INTRODUCTION

The Baltimore reduced-intensity conditioning (RIC) regimen using high-dose post-transplant cyclophosphamide (PTCY) is considered as a standard of care for haploidentical allogeneic stem cell transplantation (allo-SCT). However, it is associated with relatively low survivals (1-year and 2-year overall survival (OS): 46 and 36%; 1-year and 2-year event-free survival (EFS): 34 and 26%) and a high incidence of relapse (51% and 58% at 1 year and 2 years), especially when considering myeloid malignancies (<15% of 2-year EFS) [1] Recently, more intensive conditioning regimen (myeloablative or reduced-toxicity myeloablative) have been proposed to improve the outcome of patients with haplo-identical donors, but this could be applied only in selected younger individuals with no co-morbidities [2]. Indeed, inferior survival has been reported for older patients receiving a myeloablative haplo-transplant compared to the use of RIC regimens [3].

Clofarabine is a second-generation purine analogue with both anti-myeloid and anti-lymphoid leukemic activity [4]. Recently, clofarabine has been used as part of fludarabine-busulfan (Bu)-based conditioning regimen instead of fludarabine. This showed very promising results with a good safety profile, both in myeloablative (CloBu4) [5] and RIC (CloBu2) [6] settings. Moreover, clofarabine also appears to have more anti-myeloid activity than fludarabine after allo-SCT [5, 7]. Here, we hypothesized that replacing fludarabine by clofarabine as part of the Baltimore regimen (Clo-Baltimore regimen) could improve the outcome of patients with (mainly) haplo-donors and myeloid malignancies ineligible for myeloablative transplant.

## RESULTS

### Characteristics of patients

Between March 2014 and March 2017, 36 adults (males *n =* 18; median age: 60.5 years old) received the Clo-Baltimore regimen in our institution. The majority of cases suffered from acute myeloblastic leukemia (AML, *n =* 17) or myelodysplastic syndrome (MDS, *n =* 13). Twenty-one of them were in complete remission (CR) at transplant (AML: CR1 *n =* 12; CR2 *n =* 4; CR3 *n =* 1; MDS: CR1 *n =* 3; plasmacytoid dendritic cell neoplasm: CR1 *n =* 1), while 15 patients were transplanted with active disease (MDS *n =* 10, myelofibrosis (MF) *n =* 3; chronic myeloid leukemia *n =* 1, mixed MDS/myeloproliferative syndrome *n =* 1). Active MDS was defined by bone marrow blasts comprised between 5 and 10% at transplant. The disease risk index was retrospectively assessed according to Armand *et al.* [8]. The types of donor were haplo-identical (*n =* 27), sibling in 6, matched unrelated in 2 and 9/10 mis-matched unrelated in 1. Eight patients (AML *n =* 1; MDS *n =* 4; MF *n =* 2; mixed syndrome *n =* 1) received a graft from a matched donor (sibling *n =* 2, unrelated *n =* 6) at the discretion of the physician. Characteristics of the patients are given in Table 1.

**Table 1:** Patients characteristics

*N =* 36; Period: March 2014–March 2017	*N* (%)
Gender : male	18 (50%)
Median age : years (range)	60.5 (31–70)
Disease Acute myeloid leukemia Myelodysplastic syndrome Myelofibrosis Chronic myeloid leukemia Myelodysplastic syndrome/myeloproliferative disease Plasmacytoid dendritic cell neoplasm (pDC)	17 13 3 1 1 1
Disease status Complete remission (CR) 1 CR2 CR3 Active disease	16 4 1 15
Previous allograft	7
Disease risk index [8] Low Intermediate High Very high Not applicable	2 18 14 1 1 (pDC)
HCT-CI score [23] 0 1 2 3 4 or more	3 2 11 15 5
Donor Median age: years (range) Haplo-identical (son, daughter, sister, brother, father, nephew) Sibling Matched unrelated Mismatch unrelated 9/10	44 (23–71) 27 (10, 2, 7, 4, 2, 2) 6 2 1
CMV status recipient/donor: −/− −/+ +/+ +/−	20 4 8 4
ABO compatibility Compatibility Minor incompatibility Major incompatibility	20 10 6
Peripheral Blood Stem Cell graft Median CD34+ cells/kg	36 7.09 (1.45–12.11)

### Outcomes

Except for one patient (a 60-year old female with active MDS, obesity and diabetes mellitus, haplo-donor) who died during aplasia of multiple organ failure after sepsis, all patients engrafted. The median time of neutrophils (>1 × 10^9^/L) and platelets (>50 × 10^9^/L) recoveries were 18 (range: 8–27) and 28.5 (range: 11–111) days, respectively. The median number of red cell and platelet transfusions per patient during aplasia were 9 (range: 0–32) and 10 (range: 2–69), respectively. Full median donor chimerism was observed at each point evaluated between day+30 and +90/100 (peripheral blood: 99.9% (range: 41.5–100) at day+30, 99.9% (range: 24–100) at day+60 and 99.9% (range: 43–100) at day +90/100;CD3^+^ T-cells: 99.9% (range: 23–100) at day+60 and 99.9% (range: 43–100) at day+90/100.

**Figure 1:**
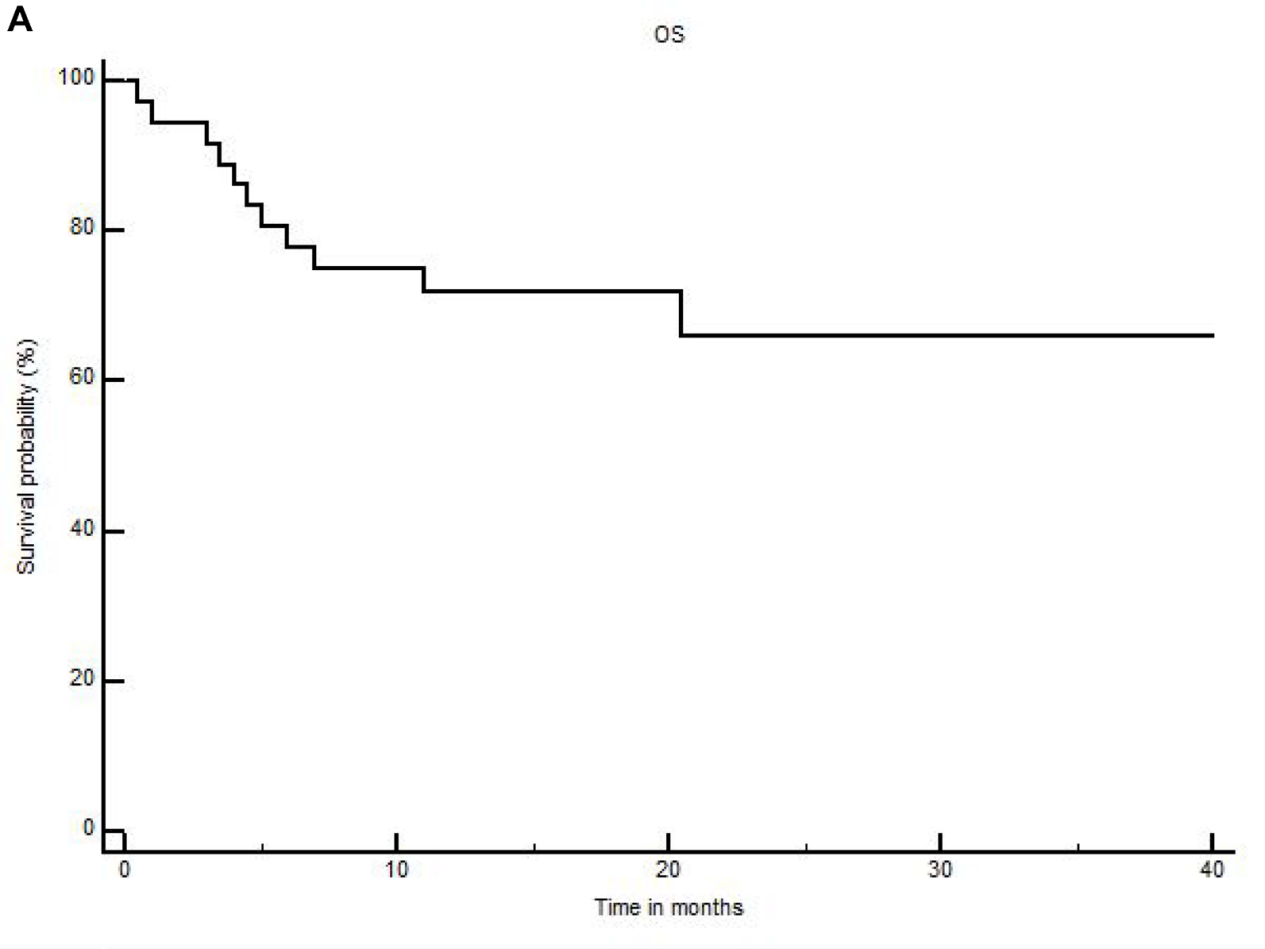

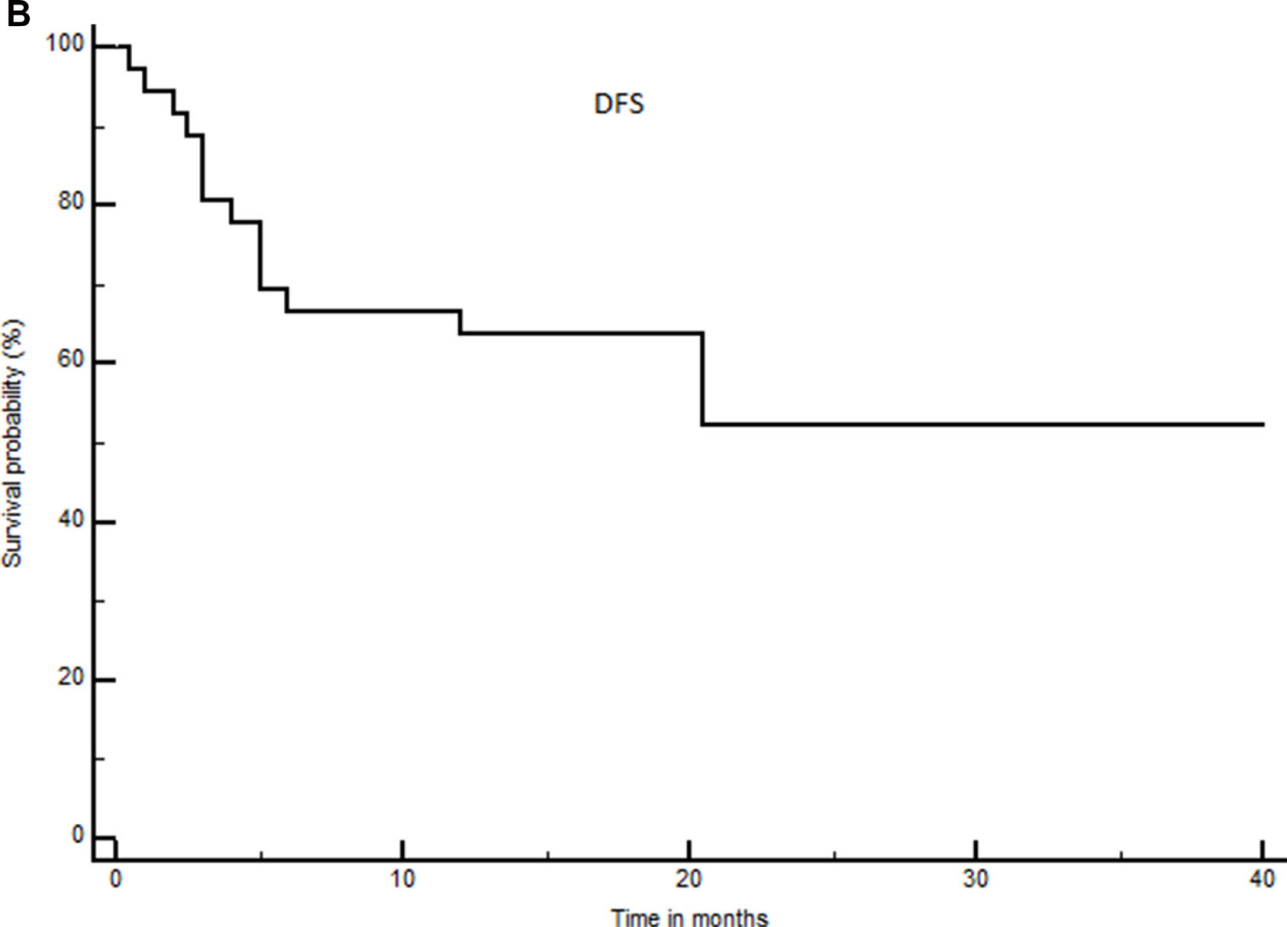
Overall survival (**A**) and disease free survival (**B**) for the whole cohort.

With a median follow-up of 18 months for patients alive (range: 7–47), 18-month and 2-year OS were 72 ± 7.5% and 66% ± 9% (Figure 1A), while 18-month and 2-year DFS were 63.8 ± 8% and 52.2 ± 10% (Figure 1B), respectively. Non-relapse-mortality (NRM) at 100 days, 1 year and 18-months were 5.5%, 11.1% and 11.1%, respectively. Eleven patients have relapsed so far, with a median time between allo-SCT and relapse of 4 months (range: 2–46), including one very late relapse. The incidence of relapse at 1-year and 18-months were both 25%. The incidences of grade 2–4 and grade 3–4 acute GVHD (1 patient non evaluable) were 48.5% and 8.5% at day+100, respectively (grade 2 cutaneous *n =* 9; gut *n =* 5, grade 3 gut *n =* 1; cutaneous *n =* 1; grade 4 gut *n =* 1). The incidence of 18-months chronic GVHD (considering patients alive after day+100 *n =* 33) was 21% (mild 6% and moderate+severe 15%, including the case who received only one day of PTCY). Grade 2–4 and grade 3–4 acute GVHD rates at day+100 were 56% and 11.1% for patients transplanted with mis-matched donors vs 25% and 0% for cases receiving a graft from a matched donor, respectively. Moreover, when excluding the patient who received only one day of PTCY, 18-months moderate/severe chronic GVHD was 14.2% in the mismatched donor group vs 0% in the matched group. GRFS rates at 18-months and 2-years were 52.6 ± 8% and 40.9 ± 9%, respectively. Seven patients received donor lymphocyte infusions (DLI) at doses comprised between 1 × 10^5^ and 5 × 10^6^ CD3^+^/kg, for mixed chimerism (3 MF, 1 patient obtained full chimerism), persistent positive minimal residual disease (1 CML and 1 AML, both patients obtained negative MRD) or as relapse prevention because of a high-risk disease with complex caryotype (1 MDS patient still in CR at 17 months and 1 AML patient still in CR at 22 months post-transplant). Only one patient developed chronic GVHD after DLI. At last follow-up (February 2018), 11 patients have died, the causes of death being relapse in 6, dermatomyositis in 1, sepsis in 3 and grade 4 acute GVHD in 1. All patients died within 6 months post-transplant, except the patient who died from dermatomyositis at 20.5 months post-transplant. The diagnosis of dermatomyositis was difficult in this 60-year old woman allografted for a high-risk myelodysplasia in first complete remission. She didn’t developed acute or chronic GVHD patient after transplant and developed dermatomyositis without any pre-existing signs of this disease or autoimmunity. It was considered as a paraneoplastic syndrome but no evidence of malignancy was documented.

### Univariate analysis

In univariate analysis, there were no differences regarding 18-month survivals between patients allografted: i) for AML vs MDS (OS 70 ± 11% vs 69.2 ± 13%, *p* = 0.3; DFS 64.7 ± 11% vs 61.5 ± 13%, *p* = 0.65), or ii) with haplo-identical vs other donors (OS: 66.2 ± 9% vs 88.8 ± 10.4%, *p* = 0.16; DFS 59 ± 9.5% vs 77.8%, *p* = 0.6). When comparing intermediate vs high+very high DRI patients, similar relapse incidence (26% vs 22%, *p* = 1, DFS (*p* = 0.96) and OS (*p* = 0.45) were observed between both groups.

### Infections post-transplant

One patient developed a mucormycosis infection with a favorable outcome under isavuconazole. No aspergillosis was documented. Overall, HHV-6 reactivation occurred in 67% of patients while 2 patients required rituximab for EBV reactivation and 9 patients antiviral treatment for CMV reactivation. Only one CMV disease was documented (colitis). Two patients developed adenovirus infection and 10 had symptomatic BK virus cystitis, including 1 with hemorrhage. HHV-6 infection was detected in 24 (66.6%) out of the 36 patients included in the study, but no HHV6 disease was documented.

### Other toxicity

One patient had a severe PTCY-related myocarditis occurring 10 days after the first dose of PTCY with a FEV at 15%. This 68-year old female allografted for a myelodysplasia with no previous cardiac events or chemotherapy treatment was transferred in intensive care unit for one month and recovered ad integrum. She’s still alive at 18 months post-transplant with normal cardiac function. Also, no veno-occlusive disease was observed in this study. Only cytokine release syndrome <= grade 2 were observed in this series (*n =* 10), both after haplotransplant (*n =* 7) or matched transplant (*n =* 3).

## DISCUSSION

Except sequential approaches for refractory hematological malignancies, [9–12] to our knowledge, there is no series, so far, reporting the use of a clofarabine-based RIC regimen with PTCY. Here, the Clo-Baltimore regimen showed very encouraging results for patients with myeloid malignancies both in CR and with active disease at transplant. A lower rate of relapse was also documented, although a non myeloablative conditioning regimen was used, which is suitable for older patients and subjects with co-morbidities. This confirms the high anti-myeloid leukemic activity of clofarabine as part of a RIC regimen including not only haplo-identical but also matched/mismatched donors. These results also compare favorably with studies reporting results of other RIC regimens in the haplo-myeloid setting [3, 13].

PBSC as stem cell source and ciclosporin instead of bone marrow and tacrolimus, respectively, were chosen here because of our long experience of these both modality treatments as part of clofarabine-based RIC allotransplant [6]. The incidence of acute GVHD was relatively high in our series, probably due to the use of PBSC. Clofarabine may have also participated to this high incidence, as it is known to induce mucositis [14], then contributing to potentially exposing self-antigens to donor T lymphocytes. This may lead to increased acute GVHD. However, in a previous study comparing clofarabine vs fludarabine-based RIC regimen we have shown that incidence of acute GVHD were similar between both groups [7]. There is some concern regarding the use of PBSC as source of graft in the haploidentical setting, because of this higher incidence of acute or chronic GVHD [15, 16]. However, in the series by Ruggeri *et al.*, [15] engraftment was higher with PBSC (95% vs 92%, *P* < 0.001) while NRM was similar between PBSC and bone marrow (BM). In the series by Bashey *et al.*, [16] the median times to neutrophil and platelet recovery were slower after transplantation of BM compared with PB (17 v 16 days for neutrophils, *P* <. 001; and 26 v 25 days for platelets, *P* =.03) while again NRM was similar between both groups. Of note, NRM at day 100 and 1 year (<6% and 11%) were in the range of data published previously [17, 18], suggesting a low toxicity of our procedure. Similar survivals have been documented also using either PBSC or BM but a lower incidence of relapse have been reported using PBSC for haploidentical alloSCT, especially in leukemic patients [16]. Here the use of PBSC for each patient may explain also the could results regarding the relatively low incidence of relapse overall.

Although this is a small cohort, the results are very interesting for patients with matched donors, not only in terms of survivals but also regarding the incidence of GVHD, even using PBSC. The role of PTCY is probably determinant and its use as a sole GVHD prophylaxis has been already proven to be efficient in the myeloablative setting with matched donors [19, 20]. We are currently testing this hypothesis with the Clo-Baltimore approach for matched donors as well (https://clinicaltrials.gov/ # NCT03263767).

The role of DLI is still controversial after haploidentical allo-SCT because of a suspected higher risk of GVHD occurrence. However, lower doses of CD3^+^ T-cells are generally infused [18]. Surprisingly, we observed a very late relapse in our cohort, suggesting that a preventive treatment for disease re-occurrence could be appropriate after a Clo-Baltimore regimen-conditioned Allo-SCT, as reported here. Prophylactic DLI associated or not to 5-azacytidine may help improve patients outcomes [21, 22].

In conclusion, the Clo-Baltimore RIC regimen appears to be an effective platform for older patients with both haploidentical or matched donors and should be considered in the future for prospective and comparative studies in allo-SCT.

## MATERIALS AND METHODS

### Study design

This retrospective study included all adults (>=18 years old) who received a Clo-Baltimore regimen for myeloid malignancies at the University Hospital of Nantes. All patients had contra-indications to myelo-ablative conditioning regimen. Use of the Clo-Baltimore regimen instead of the Baltimore regimen started in 2014 in our department when considering haplo-identical transplantation for myeloid hematological diseases because of our good experience with clofarabine in the matched setting [6]. All patients provided informed consent for data collection before the allo-SCT. This retrospective study was approved by the Ethics Hematology review board of the CHU of Nantes.

### Conditioning regimen, source of graft and supportive care

**Figure 2:**
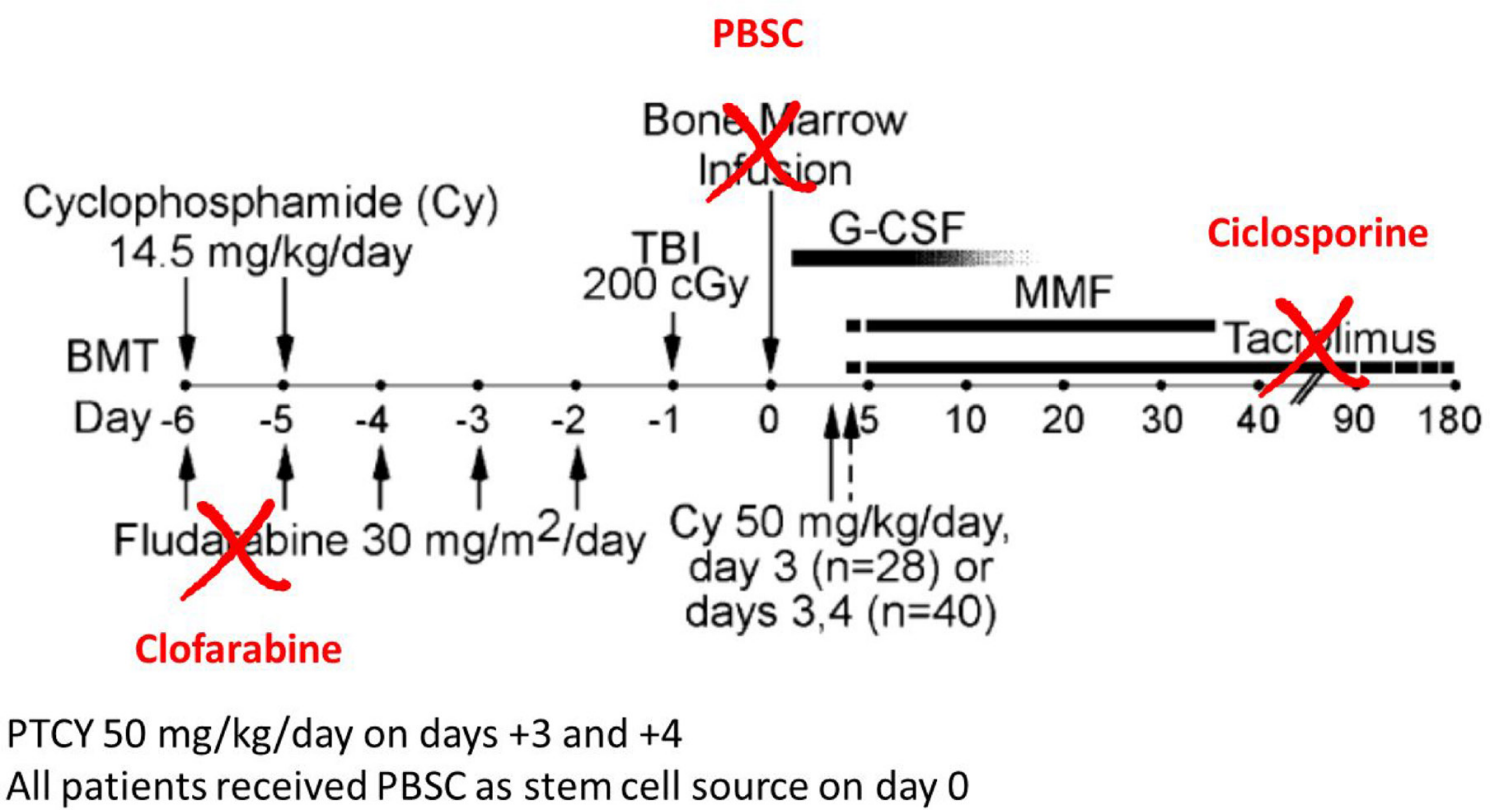
Differences between the Baltimore regimen and the Clo-Baltimore regimen used in the study (adapted from Luznik *et al*. [1]) Abbreviations: PBSC: peripheral blood stem cells; PTCY: post-transplant cyclophosphamide

The Clo-Baltimore regimen (Figure 2) consisted of Clofarabine 30 mg/m^2^/day on days -6 to -2, cyclophosphamide 14.5 mg/kg on day -6, low dose total body irradiation 2 Grays on day-1. All patients received PTCY 50 mg/kg/day on days +3 and +4, except one who had a matched donor and received only one day of PTCY. Further GVHD prophylaxis consisted in cyclosporine A and mycophenolate mofetil initiated on day +5. G-CSF was also administered systematically from day+5 on, until the end of aplasia. All patients received PBSC as stem cell source on day 0, fluconazole as fungal prophylaxis and i.v. immunoglobulins once a week until day+100 as infection prevention.

### Statistical analyses

The clinical outcomes studied were 18-month and 2-year overall survival (OS), disease-free survival (DFS), relapse incidence (RI) and non-relapse mortality (NRM). OS was defined as the time from day 0 of allo-SCT to death or last follow-up for survivors. DFS was defined as time from day 0 of allo-SCT to time without evidence of relapse or disease progression censored at the date of death or last follow-up. Relapse was defined as any event related to re-occurrence of the disease. NRM was defined as death from any cause without previous relapse or progression. Probabilities of OS and DFS were calculated using the log-rank test and Kaplan–Meier graphical representation. Acute and chronic GVHD were diagnosed and graded according to standard criteria [24, 25] The GVHD-free/relapse-free survival (GRFS), defined as alive with no previous grade III–IV aGvHD, no moderate or severe chronic GVHD and no relapse [26], was also studied. Univariate analyses were performed using the log rank test for OS and DFS. A *p* value < 0.05 was considered as statistically significant. Analyses were performed using the Medcalc^®^ software (Ostend, Belgium).
